# Comparative accuracy evaluation of patient-specific 3D-printed guide and neuronavigation for ventriculoperitoneal shunt in dogs: a dual-phase *ex vivo* and *in vivo* study

**DOI:** 10.3389/fvets.2025.1665844

**Published:** 2025-11-14

**Authors:** YoungJin Jeon, Cheongwoon Cho, Jaemin Jeong, Björn Meij, Haebeom Lee

**Affiliations:** 1 Department of Veterinary Surgery, College of Veterinary Medicine, Chungnam National University, Daejeon, Republic of Korea; 2 Department of Clinical Sciences, Faculty of Veterinary Medicine, Utrecht University, Utrecht, Netherlands

**Keywords:** hydrocephalus, ventriculoperitoneal shunt, ventricular catheter, patient-specific guides, neuronavigation

## Abstract

**Background:**

Ventriculoperitoneal shunting (VPS) is an effective treatment for canine hydrocephalus, but complications related to ventricular catheter (VC) misplacement remain a concern. Although neuronavigation improves accuracy, its cost and complexity limit veterinary use. Patient-specific 3D-printed guiding systems (PS-3DGS) offer a potential alternative.

**Objectives:**

To compare the accuracy and feasibility of PS-3DGS compared to electromagnetic neuronavigation for VC placement in 3D-printed canine cranio-ventricular models (CVMs) and Beagle dogs.

**Animals and study design:**

Ten 3D-printed CVMs (*ex vivo* study) and five experimental Beagle dogs (*in vivo* study).

**Methods:**

VC placement was performed using PS-3DGS and neuronavigation in CVMs and experimental animals. Accuracy was assessed by the distance from the VC tip to the foramen of Monro (DFM), tip coordinates (X, Y, Z axes), tip placement error, intraventricular insertion length (IIL), and catheter contact with the ventricular wall (VVL). Intraoperative procedural time and anatomical variables, including skull and cranial indices, were also analyzed.

**Results:**

PS-3DGS showed no significant difference in DFM compared to neuronavigation in CVMs and Beagle dogs. PS-3DGS achieved significantly lower VVL and reduced procedural time than neuronavigation in the CVM study (*p* = 0.011 and *p* = 0.039, respectively). In dogs, DFM with PS-3DGS was comparable to both neuronavigation and the *ex vivo* results. Entry point error correlated negatively with cranial index (r = −0.9, *p* = 0.037).

**Conclusion:**

PS-3DGS provided accuracy comparable to neuronavigation while simplifying the procedure. It represents a viable, cost-effective alternative for canine VPS surgery, potentially enhancing catheter placement and reducing complications.

## 1 Introduction

The ventricular system of the brain is a complex network of interconnected cavities that produce and circulate cerebrospinal fluid (CSF), which is essential for maintaining intracranial pressure, providing nutrients, and removing metabolic waste. This system includes the paired lateral ventricles, third ventricle, and fourth ventricle, connected by passageways such as the interventricular foramen and mesencephalic aqueduct. CSF circulates through the ventricles and is absorbed primarily by the arachnoid villi (1). Any disruption to this flow—whether due to blockage, impaired absorption, or overproduction of CSF—can lead to hydrocephalus, an abnormal accumulation of CSF within the ventricles. Hydrocephalus can be congenital or acquired, with congenital hydrocephalus particularly prevalent in small dog breeds such as Chihuahuas and Maltese (2–4).

In both human and veterinary medicine, ventriculoperitoneal shunt (VPS) placement is the standard surgical treatment for hydrocephalus when medical management fails (4, 5). The VPS drains excess CSF driven by gravity differences from the cerebral ventricles to the peritoneal cavity, thereby reducing intracranial pressure and ventricular volume (4, 6–8). VPS can also be used palliatively to relieve intracranial pressure secondary to intracranial tumors (9–11). However, the success of shunting heavily depends on accurate placement of the ventricular catheter (VC) within the ventricle; an improperly placed catheter can lead to shunt malfunction. Inaccurate catheter placement is associated with complications such as ventricular obstruction, underdrainage or mechanical failure, which significantly affect patient outcomes (12–15). Reported overall complication rates range from approximately 22 to 31.6%, with most complications occurring within the first 3 months after surgery (6, 8, 16, 17).

Over the years, advancements in neurosurgical technique have sought to improve the precision and safety of VC placement. Technologies such as ultrasonography, neuronavigation, neuroendoscopy, and intraoperative imaging have been developed to enhance accuracy during VPS placement (18, 19). Despite their effectiveness, these systems require specialized and expensive equipment, increasing surgical complexity and cost. Consequently, there is growing interest in simpler, cost-effective solutions that can maintain or even improve placement accuracy without the need for such equipment. One promising alternative is the use of patient-specific 3D-printed guiding systems (PS-3DGS) that offer a tailored approach to catheter placement by utilizing preoperative imaging to design and fabricate a guide fitted to the patient’s unique cranial anatomy. In human medicine, several studies have demonstrated that PS-3DGS has the potential to achieve feasibility and accuracy while bypassing the high costs and technical demands of advanced navigation equipment (20–22).

Given the need for improved accuracy and the limited access to neuronavigation in veterinary neurosurgery, we sought to determine whether a patient-specific 3D-printed guide could provide ventricular catheter placement accuracy comparable to that of electromagnetic (EM) neuronavigation. To address this question, we designed a two-phase experimental study. In the first phase, an *ex vivo* investigation was performed using custom 3D-printed canine cranio-ventricular models (CVMs), allowing a direct side-by-side comparison of catheter placement accuracy between the PS-3DGS and an EM neuronavigation system under controlled laboratory conditions. In the second phase, an *in vivo* study was conducted in experimental Beagle dogs undergoing VPS surgery, to assess the feasibility and accuracy of using the PS-3DGS in actual surgical practice including the potential effects of soft tissue and operative handling on guide performance. By combining a benchtop *ex vivo* model evaluation with a translational *in vivo* trial, this study provides a comprehensive assessment of the PS-3DGS approach for VPS placement in dogs. The overarching aim was to determine if a low-cost, patient-specific guide can achieve shunt catheter placement accuracy on par with gold-standard neuronavigation, thereby potentially improving surgical outcomes and making precise hydrocephalus treatment more accessible in veterinary medicine. The hypothesis was that the PS-3DGS provides ventricular catheter placement accuracy comparable to that of EM neuronavigation in both the *ex vivo* and *in vivo* phases.

## 2 Materials and methods

### 2.1 Study design

This study consisted of two sequential experimental phases: an *ex vivo* phase using CVMs and an *in vivo* phase using experimental Beagle dogs. In the *ex vivo* phase, 10 CVMs were used for paired ventricular catheter insertions into the left and right lateral ventricles, utilizing two different methods: a PS-3DGS and EM neuronavigation. Each method was applied five times per side, resulting in a total of 10 insertions per method (23). In the *in vivo* phase, five purpose-bred, healthy adult Beagles (2 neutered males and 3 spayed females; mean body weight 8.3 ± 0.68 kg) were used, with each dog receiving one catheter via PS-3DGS and one via EM neuronavigation. The sample size was determined to meet ethical standards by minimizing animal use and was based on an *a priori* power analysis conducted using effect size data from the *ex vivo* study. All experimental procedures were approved by the Institutional Animal Care and Use Committee of Chungnam National University (approval no. 202410A-CNU-208). Following the study, all dogs were returned to the institutional research colony, where they were monitored for health and welfare in accordance with animal care guidelines.

### 2.2 Cranio-ventricular model construction

The cranio-ventricular models were generated from pre-existing computed tomography (CT) scans of canine heads. Five CT scan datasets were obtained from clinical dogs that had presented to a veterinary hospital (four Pomeranians and one Maltese, representing dogs predisposed to hydrocephalus), and five were obtained from purpose-bred Beagle dogs. All CT scans (Aquilion prime SP, Canon medical systems corp., Otawara, Japan) were performed with a slice thickness of 1 mm of the skull, exported in Digital Imaging and Communications in Medicine (DICOM) format. From each CT dataset, 3D models of the skulls and lateral ventricles were created using 3D Slicer software (v4.11; Surgical Planning Laboratory, Brigham and Women’s Hospital, Boston, MA, USA). Semi-automated thresholding and manual editing were used to segment the bony skull and the ventricular system. A 1 mm spherical indicator was used to mark the level of the foramen of Monro and to serve as a target reference.

The skull model was then imported into modeling software (Autodesk 3ds Max 2019, Autodesk, San Francisco, CA, USA) for further processing. Key cranial measurements—skull length (prosthion to inion), skull width (zygomatic width), cranial length (nasion to inion), and cranial width (neurocranial width)—were taken on the 3D model to calculate the skull index (SI = skull width/skull length × 100) and cranial index (CI = cranial width/cranial length × 100) for each specimen (24). These indices categorize head shape; a higher index indicates a wider, shorter skull (brachycephalic tendency) and a lower index indicates a longer, narrower skull (dolichocephalic tendency). Ventricular size was quantified on the original CT images under the soft tissue window (window level 25 and width 100) by the ventricles-to-brain ratio (VBR), defined as the height of the lateral ventricle relative to the height of the brain on a transverse CT slice at the level of the interthalamic adhesion (25).

To fabricate the CVM, the 3D skull and ventricle models were prepared for 3D printing. Each skull was virtually sectioned into rostral and caudal parts at the level of the temporomandibular joints, and the ventricular cast was connected to the skull interior by a few narrow support struts. These struts fixed the ventricle in place within the cranial vault during printing and would later be removed. The modified skull and ventricle models were printed using a stereolithography (SLA) 3D printer (Photon M3 Max, Anycubic, Shenzhen, China) with a standard photo-curable resin (Harpiks Standard, Zerone, Seoul, South Korea) with a layer thickness setting of 0.1 mm and an exposure time of 2 s. After printing, the models were washed with 99% isopropyl alcohol using ultrasonic cleaner and cured in LC-3DPrint Box (NextDent, Soesterberg, Netherlands) for 5 min.

**Figure 1:**
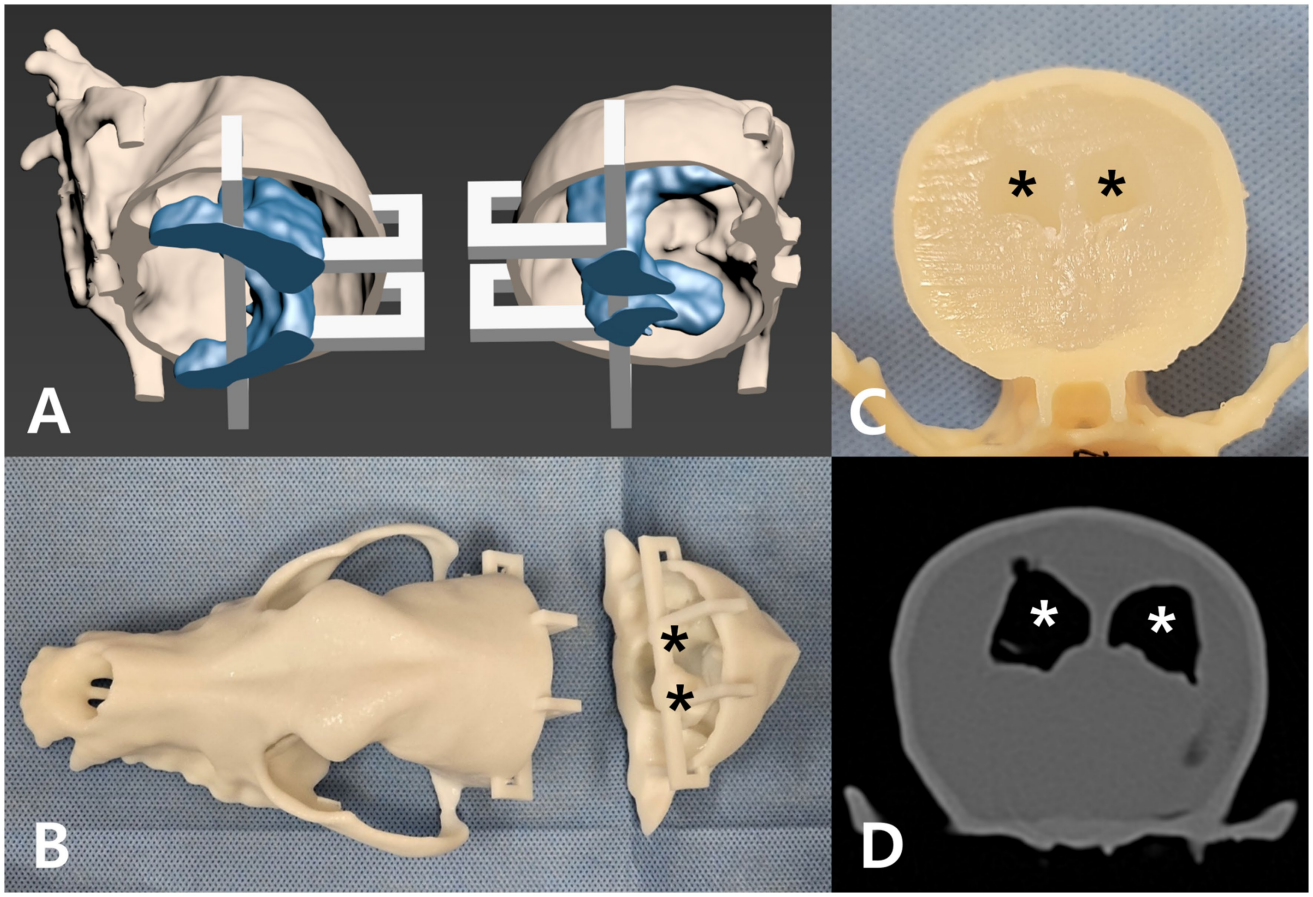
Development of the cranio-ventricular model (CVM). **(A)** 3D-reconstructed dog skull (beige) and ventricular system (blue) with a detachable section (white supports) for later agar filling. **(B)** The skull and ventricle models 3D-printed in resin. Black asterisks mark the lateral ventricles in the caudal skull. **(C)** Agar gel filling the cranial vault after removal of the ventricle mold. Black asterisks mark the hollow lateral ventricle spaces. **(D)** Transverse CT image of the completed CVM, with white asterisks indicating the lateral ventricles.

The resulting rigid resin skull contained a hollow space in the shape of the lateral ventricles, held in place by the printed struts. To simulate brain parenchyma, a 1.5% agarose gel (Promega Agarose LE; Madison, WI, USA) was poured into the cranial cavity of each skull. Once the gel set, the resin ventricle cast and support struts were carefully removed, leaving a gel-filled cranial vault with empty ventricular-shaped cavities in the gel to represent the lateral ventricles. The two halves of the skull were then reassembled and glued (cyanoacrylate adhesive) to create a complete head model containing agarose “brain” and realistic ventricular spaces. After the complete fabrication of the CVMs, CT scans were performed to assess their anatomical reproducibility (Figure 1).

### 2.3 Patient-specific 3D guide design

**Figure 2:**
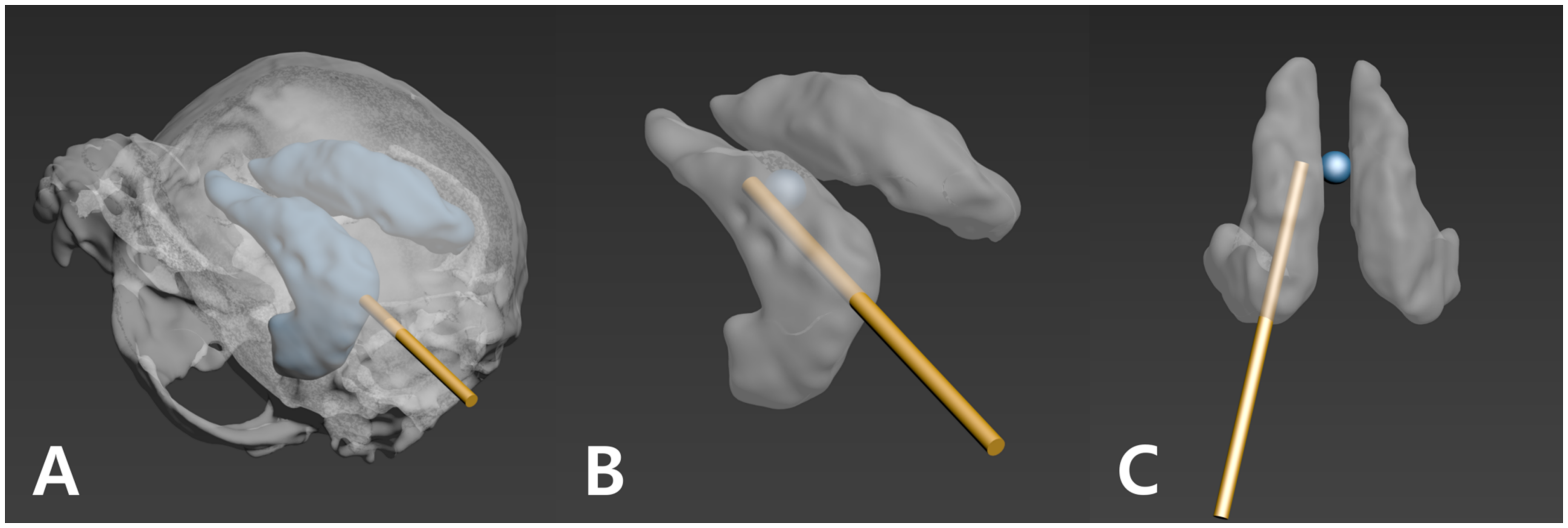
Three criteria for planning the ventricular catheter trajectory. A 1.2 mm gold cylinder was used to simulate the catheter path during planning. **(A)** The trajectory is directed away from the ventricle wall (the gold cylinder is not visible through the ventricle wall). **(B)** The catheter’s perforated segment is inserted as far into the ventricle as possible. **(C)** The catheter tip is positioned as close as possible to the foramen of Monro (blue sphere; reference point).

A custom 3D-printed guide was designed for each CVM to direct the ventricular catheter insertion. The guiding system comprised two interlocking components—an anchor guide that attached to the dog’s upper jaw (maxillary teeth), and a midline guide that fit over the dorsal midline of the skull—together providing a stable trajectory for the catheter. Guide design was performed using 3ds Max software. First, a virtual surgical plan was created by positioning a cylindrical rod (2.1 mm diameter, representing the catheter) in the 3D model of the ventricle and skull. The rod’s placement followed three key criteria: (1) the catheter tip as close as possible to the foramen of Monro (close to the reference 1 mm cylinder), (2) the entire 16 mm perforated tip segment residing inside the lateral ventricle, and (3) the catheter trajectory avoiding contact with ventricular walls (Figure 2). Using these criteria, an optimal entry point on the skull was determined (generally on the parietal bone, near both the external sagittal crest and the nuchal crest) such that the catheter would pass through the ventricle along its longitudinal axis (laying within the ventricular body) from a caudal-to-rostral direction.

**Figure 3:**
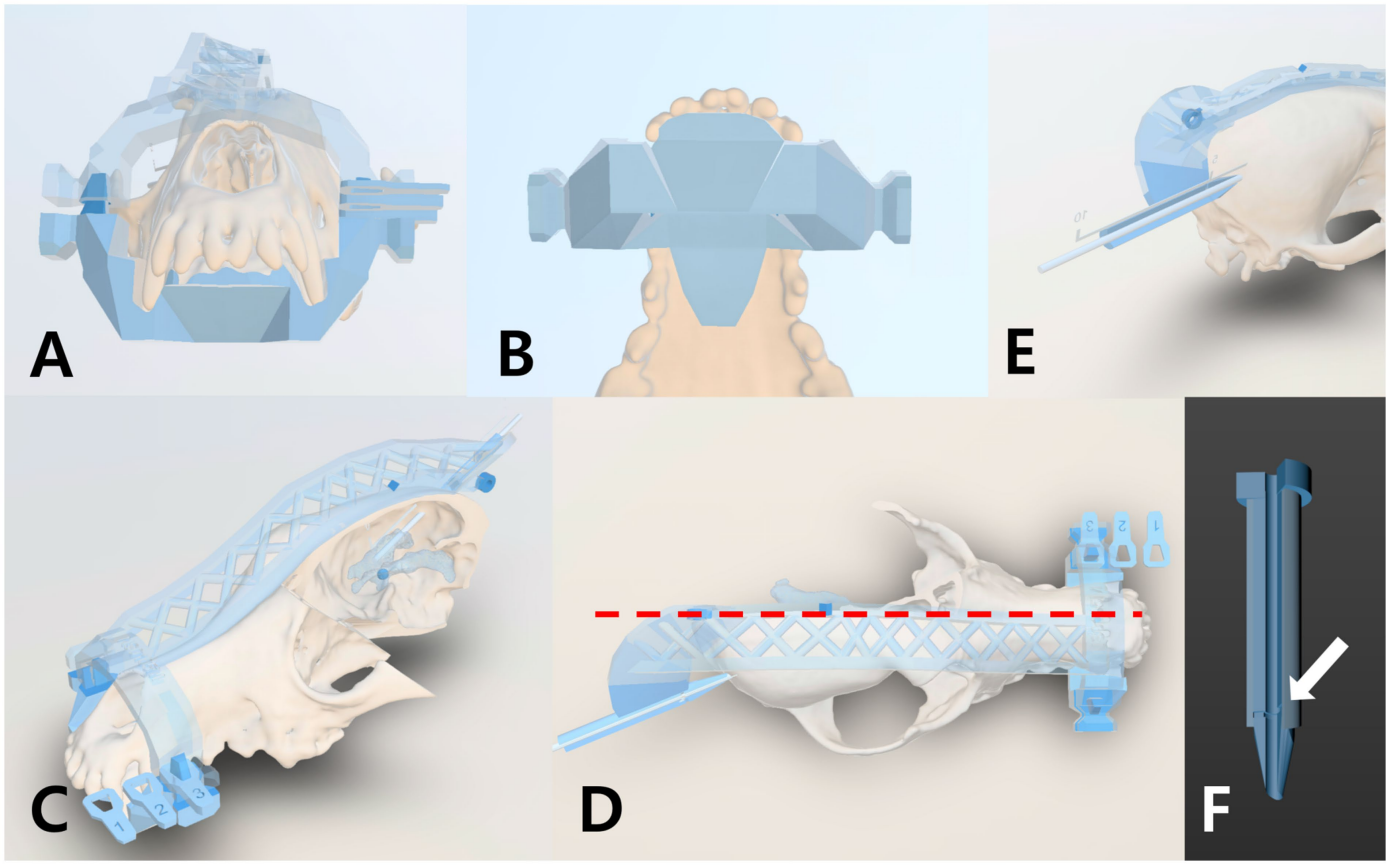
Design Features of the Patient-Specific 3D Guiding System. The system consists of an anchor guide and a midline guide. **(A)** Front view of the anchor guide, which fits over the first premolar and canine teeth on both sides. **(B)** Ventral view of the anchor guide in contact with the canine teeth and surrounding palatine bones. **(C)** Oblique front view of the midline guide contoured to the nasal, frontal, and parietal bones. **(D)** Dorsal view of the midline guide aligned with the skull midline (red dash line). **(E)** The posterior end of the midline guide functions as a sliding support, with its tip maintaining contact with the bone surface. **(F)** A marking line (white arrow) on the guide aligns with the 5 cm mark on the catheter, ensuring the planned insertion depth.

With the planned trajectory defined, the guide components were modeled. The anchor guide was shaped to cap over the maxillary canine and first two premolar teeth on both sides, using the dental arcades as a stable support (since the teeth are rigid and fixed in the skull). The midline guide was designed to conform to the dorsal midline of the skull, extending from the nasal bones over the frontal and parietal bones, and ending near the occiput. The two guide pieces were engineered to snap together via matching convex/concave blocks so that, when joined, the midline guide sat in a precise orientation relative to the anchor (and thus the skull and jaw). The midline guide contained an integrated gliding section—essentially a hollow cylindrical tunnel—aligned along the planned catheter trajectory. This section had an internal diameter of 2.3 mm and an outer diameter of 5 mm, with a lateral opening spanning 120° of the circumference to facilitate catheter insertion and removal. At the proximal end of the gliding tunnel, a negative marker was included to indicate the correct catheter insertion depth; this marker corresponded to the first 5 cm marking on the catheter and aligned with a negative engraved line on the guide when the catheter was fully seated. This ensured the catheter was inserted to the pre-planned length (Figure 3). For additional strength during printing, temporary support bridges were added connecting portions of the guide to be removed after fabrication. All guides were printed on the same SLA printer with a biocompatible, transparent resin (Clear SG, Dentis, Daegu, South Korea) that is classified as a Class II medical device, which was approved for surgical guide fabrication. Printing and post-processing (washing, UV curing) parameters were the same as for the CVMs.

For the *in vivo* Beagle study, an additional consideration was included: the effect of soft tissue. A soft tissue layer was reconstructed individually for each dog from its own CT DICOM using 3D Slicer, and this patient-specific layer was used to contour the contact surfaces of the guide. The anchor guide was further shaped to fit not only the teeth but also the hard palate and gingiva. The midline guide was contoured to align with the soft tissue layer over the midline skull (nasal, frontal, and parietal bones), except for the distal end of the gliding section, which was kept in contact with the underlying bone to secure the entry trajectory. This hybrid approach, integrating both skeletal and soft tissue contours, was intended to improve seating accuracy of the PS-3DGS in the Beagle dogs, while maintaining the trajectory stability required for precise catheter placement.

### 2.4 Ventricular catheter and equipment

In both phases of the study, the same type of VC and surgical instruments were used to ensure comparability. The VC was a 23 cm length commercial canine shunt catheter (model no. 41207, Minneapolis, MN, USA) with an outer diameter of 2.1 mm and inner diameter 1.2 mm. The distal tip of the catheter is perforated with multiple side-holes over a length of 16 mm. Three equidistant marker bands are present on the catheter (at 5, 10, 15 cm from the tip) to gauge insertion depth. In addition, the VC set includes a right-angle retaining clip, which is inserted into the burr hole in the skull and bent at 90° so that, when the VC is secured within it, the catheter is securely fixed in close contact with the skull surface. The set also contains a basic metal stylet with a length and diameter matching the dimensions of the VC; the stylet is inserted in the VC and provides some rigidity to the VC during insertion.

### 2.5 EM neuronavigation and equipment

For the neuronavigation method, an EM navigation system (StealthStation S8, Medtronic) was used. The system’s components included a field generator (EM transmitter), a tracked pointer probe, and an EM-tracked stylet that can be inserted into the lumen of the catheter to guide it. The navigation software allows importing the patient’s CT images and planning a trajectory, then provides real-time tracking of instruments relative to the patient after registration. All setup and use of the navigation system were performed according to the manufacturer’s guidelines.

### 2.6 *Ex vivo* procedure

Two investigators performed the *ex vivo* placement trials: one acted as the surgeon performing burring and catheter insertion, and another managed the neuronavigation workstation when applicable. Prior to intervention, each CVM was positioned and secured in sternal recumbency on the surgical table using a vacuum stabilization bag and tape to simulate how an experimental dog would be positioned.

#### 2.6.1 Using PS-3DGS

The anchor portion of the 3D guide was first seated onto the maxillary teeth of the CVM. Next, the midline guide was attached to the anchor and carefully lowered onto the skull, ensuring precise alignment with the mid-sagittal plane by confirming symmetry along the nasal bone and the occipital protuberance. With the assembled guide in place, a surgical marking pen was used to mark the entry point on the skull through the open end of the gliding tunnel. The midline guide was then temporarily removed from the anchor and shifted aside to create working space for burring. A burr hole was made at the marked entry point using a 3 mm burr bit in an electrical motor unit. After burring, the midline guide was returned to its position on the anchor. The ventricular catheter with its stylet was then inserted through the guide’s tubular gliding section until the negative line marker on the guide aligned with the catheter’s 5 cm marker, indicating full planned insertion depth. The catheter passed smoothly through the cranium, and entry into the hollow ventricular system was confirmed by a sudden loss of resistance, as the agarose gel, representing brain tissue, provided resistance until the catheter tip entered the empty space of the ventricle. Additionally, a syringe was attached to the catheter hub to check for the absence of back-pressure, since improper placement in solid gel would cause resistance when withdrawing the syringe plunger. Once correct placement was verified, the stylet was removed from the catheter. Finally, the catheter was secured by placing it in a right-angle retaining clip and then fixing both the catheter and the clip to the skull surface with medical adhesive tape.

#### 2.6.2 Using EM neuronavigation

For trials using the neuronavigation system on the CVM, the setup and procedure mimicked a clinical scenario as closely as possible. Four to five small fiducial markers (radiopaque stickers) were attached to the exterior of the model on the nasal region, between the eyes, and on the parietal surface contralateral to the planned burr hole. The number and placement of markers were scaled to the model’s size and corresponded to typical placement on an experimental dog’s head. A CT scan of the model with fiducials was then performed using identical settings as the pre-operative scans. The DICOM images were loaded into the navigation software. The CVM was placed in sternal position and stabilized as before, and an EM field generator was positioned under the position bag. An EM patient tracker (a small sensor) was affixed securely by taping under the incisor bone. The navigation system was then used to register the model’s physical space to the CT images. This was accomplished by tracing the pointer probe over the surface of the skull and touching each fiducial marker on the model while the system identified the corresponding points on the CT images. Registration was considered acceptable when the system’s reported fiducial registration error was under 2 mm (26, 27). The skin fiducials were then removed from the model to avoid interference during burring. Using the navigation software (versions 1.3.0, Medtronic), an entry point and target point were selected to replicate the same criteria that had been used for planning the PS-3DGS. Once the plan was set, the neuronavigation guided procedure was performed. The entry site on the skull was identified with the EM pointer and marked. A burr hole was drilled at this location using the same 3 mm drill. After burring, the default solid stylet of the catheter was replaced with the EM-tracked stylet. The catheter was then carefully advanced into the skull and brain model while the surgeon monitored its trajectory on the navigation screen in real-time. The goal was to align the catheter with the planned trajectory line on screen, ensuring the tip reached the ventricle target. Once the catheter was inserted to the planned depth, the stylet was removed. Correct ventricular placement was again confirmed by the free aspiration test (syringe with no resistance). The catheter was then clipped and taped to the model as done in the PS-3DGS trial. After completing both insertions, a post-procedure CT scan of the model was obtained to document catheter positions for accuracy analysis.

### 2.7 *In vivo* study

All CT scans and surgeries were performed under general anesthesia. The dogs were premedicated with medetomidine (1 μg/kg IV). After induction with alfaxalone (3 mg/kg IV), anesthesia was maintained with isoflurane (approximately 2% in 100% oxygen). The dorsal fur was clipped from the nasal and frontal regions to the occiput, as well as over a small area on the neck. To facilitate registration, six fiducial skin markers were placed on the head: over the nasal bone, between the eyes (medial canthi region), and near the external occipital protuberance and sagittal crest—areas with minimal skin movement. A thin-slice (1 mm) CT scan of the head was then performed. Each dog was carefully transported to the operating room, with particular attention paid to minimizing head movement relative to the fiducial markers by maintaining the same position on the original foam cradle used during scanning. Two investigators conducted the *in vivo* placement trials: one served as the surgeon, performing the burring and catheter insertion, while the other operated the neuronavigation workstation and positioned the PS-3DGS.

#### 2.7.1 Surgical procedure

The dogs were positioned in sternal recumbency for surgery. Each dog underwent sequential placement of two ventricular catheters—one via neuronavigation and one via PS-3DGS. To maintain accuracy of the neuronavigation system, the neuronavigation-guided placement was always performed first, followed by the PS-3DGS placement on the contralateral side. After the planning CT, the neuronavigation workstation was prepared with the dog’s images. The dog was positioned in sternal recumbency with the head flexed about 30° downward to facilitate a caudal entry point. The head was supported by a vacuum bag and secured with tape to minimize movement. The EM field generator was placed under the bag and the EM patient tracker was fixed inside the dog’s mouth, anchored across the hard palate and taped to the muzzle. The navigational registration was performed as the same sequence as the CMV study. Once registered, the skin fiducial markers were removed from the dog’s head and the surgical field was prepped.

All surgical steps were performed under aseptic conditions. A small skin incision (~2 cm) was made at the planned entry point for the first catheter, as determined by the navigation plan. Blunt dissection through the subcutaneous tissue and temporalis muscle was performed to expose the skull. The navigation pointer was used to confirm the intended entry location on the exposed cranium to account for any slight shift after muscle retraction. A 3 mm diameter burr hole was made in the skull at this site, angling the burr in alignment with the planned trajectory provided by the navigation system. An adjacent 1 mm burr hole was also made through the skull a few millimeters away, and a loop of 3–0 nylon suture was placed through this small hole—this suture would later secure the catheter’s retaining clip. The dura mater at the burr hole was cauterized and then perforated with a stab incision (11-blade) and a 24G hypodermic needle. Before inserting the catheter, all metallic instruments were removed from the field to avoid EM interference. The ventricular catheter with the EM stylet inserted was then gently advanced through the burr hole into the brain parenchyma along the planned trajectory. The surgeon tracked the catheter tip on the navigation screen in real-time, adjusting angle as needed to stay on the virtual trajectory line. When the catheter reached the target depth, the stylet was removed. Immediately, clear CSF outflow from the catheter confirmed intraventricular placement. A right-angled plastic retaining clip was positioned next to the catheter on the skull, and the catheter was slid into this clip. The previously placed nylon suture was tied around the clip, anchoring it to the skull. The excess external portion of the catheter was temporarily coiled and placed subcutaneously. The skin incision was closed with a simple continuous suture to protect the site during the second procedure. In all five dogs, neuronavigation was able to guide the catheter into the ventricle on the first attempt.

The second lateral ventricle in each dog was catheterized using the PS-3DGS. After completing the first side, the sterile PS-3DGS was brought to the surgical field. The EM tracker was removed from the mouth to eliminate encumbrance. The dog’s head remained in the same position. An assistant (not scrubbed, to handle the guide without contaminating the sterile field) positioned the anchor guide onto the maxillary teeth from the oral side. The surgeon, on the sterile field side, then took the midline guide and passed it through a small fenestration made in the sterile drape at the level of the frontal sinus. The assistant interlocked the midline guide with the anchor guide, and the surgeon gently settled it onto the dorsal midline of the skull. Proper seating was confirmed by palpation of known bony landmarks (external sagittal crest, occipital protuberance) relative to the guide’s contours. A marking pen was used to outline the planned skin incision along the path of the guide’s gliding section tip. The guides were then temporarily separated and removed from the field to facilitate approach to the cranium. A second skin incision (~2 cm) was made along the marked line for the contralateral burr hole. Muscle was split and retracted, then the guides were reassembled on the head to pinpoint the burr hole location on bone. The entry point was marked with electrocautery or surgical marker through the gliding tube. The guides were again briefly removed to avoid damage during burring. A burr hole was created and a nylon suture was placed identical to the neuronavigation side. The dura was cauterized and opened as before. The guides were then placed back on the head, fitting into their previous position. The surgeon verified the alignment one more time and then inserted the catheter through the gliding tunnel of the midline guide down to the planned depth by aligning the catheter’s 5 cm mark with the guide marker. The catheter slid in smoothly along the guide channel into the ventricle. Upon stylet removal, CSF flow confirmed success on the first attempt for all cases. The catheter was secured with the right-angled clip tied to the skull via the nylon suture, exactly as performed on the first side. The guides were then removed. The incision was closed routinely. The dog was transported to the CT suite for a post-procedure head CT to evaluate catheter placement bilaterally. Finally, the VCs were removed and skin closure was performed routinely and aseptically.

#### 2.7.2 Postoperative care

After recovery from anesthesia, the dogs were monitored in the clinic for 1 week. To mitigate potential complications from intracranial manipulation, several prophylactic treatments were administered. Levetiracetam (20 mg/kg IV over 5 min) and mannitol (0.5 g/kg IV over 15 min) were given at the end of anesthesia to reduce the risk of seizures and cerebral edema, respectively. Postoperatively, dogs received broad-spectrum antibiotics (amoxicillin-clavulanate, 12.5 mg/kg IV every 12 h) and analgesics (gabapentin, 10 mg/kg PO every 12 h; tramadol, 4 mg/kg IV every 12 h) for 5 days. The skin incisions were checked and cleaned daily. Neurologic examinations were performed daily for 14 days to monitor for any signs of deficits or complications.

### 2.8 Preoperative, intraoperative, and postoperative assessments

To evaluate the anatomical fidelity of the CVMs and the accuracy of ventricular catheter placement under both *ex vivo* and *in vivo* conditions, several quantitative and functional metrics were assessed *in silico* using 3ds Max, based on preoperative planning and postoperative CT data. These included anatomical reproducibility, catheter placement accuracy metrics, functional catheter positioning parameters. In addition to these in silico assessments, intraoperative procedural time was also measured separately for both methods to compare procedural efficiency. For these evaluations, postoperative 3D models of the skull, ventricles, and ventricular catheters were reconstructed from the postoperative CT images and rigidly superimposed *in silico* onto the original planned models by aligning consistent bony landmarks (bilateral zygomatic processes, canine teeth, and external occipital condyles). This superimposition enabled extraction of the coordinates for all distance-based accuracy evaluations. In the ex vivo phase, anatomical reproducibility of the fabricated lateral ventricles was additionally examined by comparing ventricle reconstructions from the original CT data with those of the CVMs. The overlapping volume between the original and printed ventricles was computed using a Boolean intersection tool, and reproducibility was expressed as a percentage calculated by dividing the intersected volume to the original ventricle volume.

**Figure 4:**
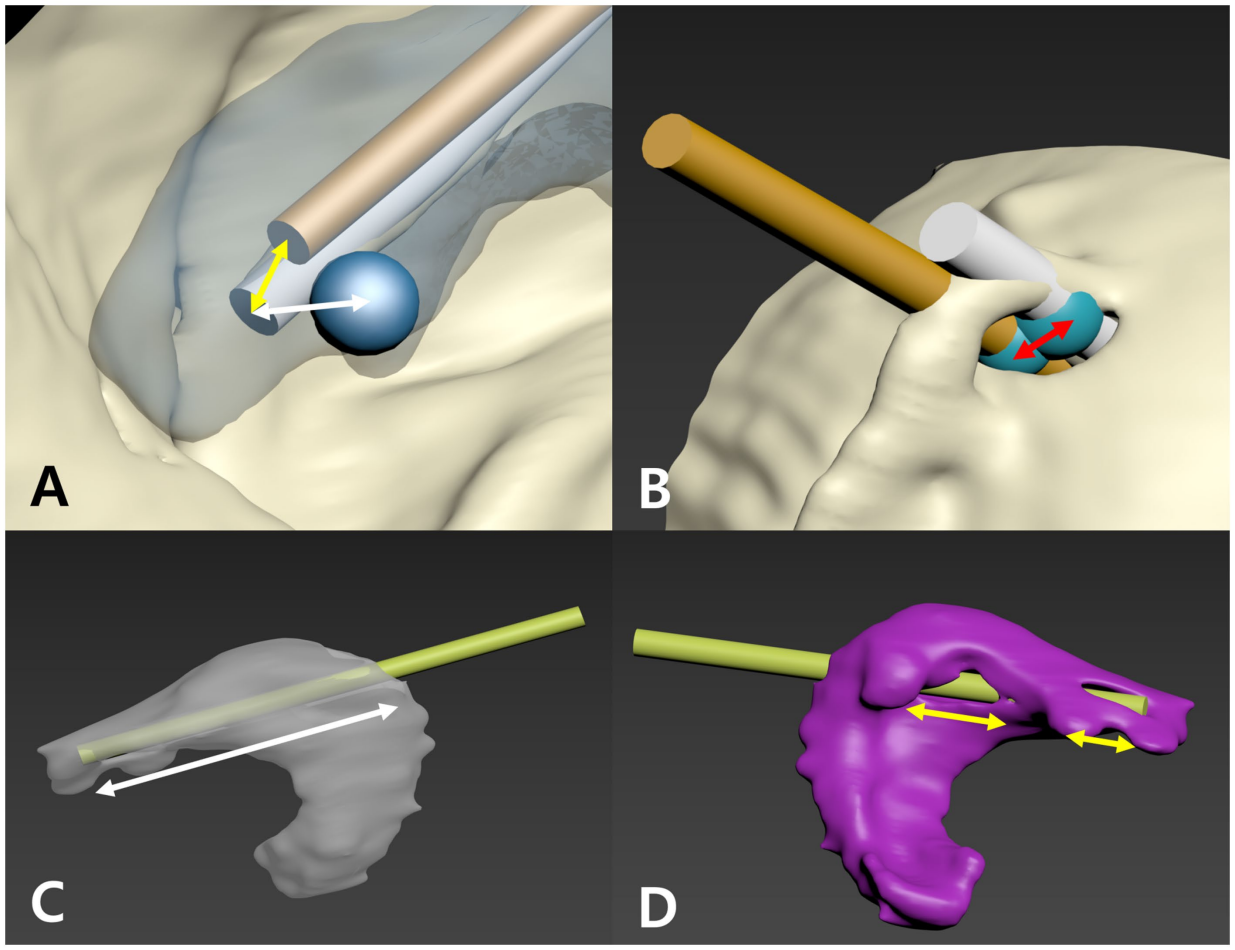
Methods for measuring parameters to assess ventricular catheter placement. **(A)** Planned trajectory (gold cylinder) and actual catheter (white cylinder) reconstructions with the foramen of Monro (FM) marked by a blue sphere. The distance from the catheter tip to the FM (white arrow) is the DFM, and the tip placement error is shown by the yellow arrow. **(B)** At the skull entry point, blue spheres mark the planned and actual catheter positions; the distance between them (red arrow) is the entry point error. **(C)** The inserted catheter (yellow-green cylinder) is used to measure the intraventricular insertion length (IIL, white arrow). **(D)** The portion of the catheter’s perforated tip in contact with the ventricular wall is measured as a fraction of the perforated tip length, defining the ventricular wall contact length (VVL).

For both the ex vivo and *in vivo* studies, the primary accuracy metric for assessing catheter placement accuracy was the distance from the catheter tip to the center of the foramen of Monro (DFM). The 3D coordinates (X, Y, Z) of the catheter tip from both the planned and postoperative CT scans, as well as the coordinates of a reference sphere (0, 0, 0) placed at the level of the foramen of Monro, were recorded (Figure 4A). In addition to DFM, several additional distance-based metrics were evaluated to comprehensively assess the accuracy and functional quality of ventricular catheter placement. For the PS-3DGS group, tip placement error—defined as the distance between the actual catheter tip and the planned tip—was measured to assess how closely the inserted catheter followed the intended trajectory (Figure 4A). Entry point error was also measured as the distance between the actual burr hole and the planned entry point on the skull (Figure 4B). All distance-based metrics in this study were calculated using the Euclidean distance formula: √((X2 − X1)^2^ + (Y2 − Y1)^2^ + (Z2 − Z1)^2^). Accordingly, DFM was calculated for both the planned and postoperative CT scans in the two placement methods. To further evaluate spatial variation, Supplementary Table S1 provides the raw data, including the absolute coordinates (X, Y, Z) of the planned and actual catheter tips, the coordinate differences (ΔX, ΔY, ΔZ), and the calculated Euclidean distance–based metrics (including DFM, tip placement error, and entry point error) for each case.

Two additional functional metrics were assessed to evaluate the quality of catheter positioning within the ventricular lumen. The intraventricular insertion length (IIL) was measured in millimeters using the measurement tool in the *in silico* 3D CT model. Specifically, it was determined as the distance from the catheter tip to the center of the catheter segment contacting the inner ventricular wall at the entrance point, representing the length of the catheter residing inside the ventricular cavity (Figure 4C). The ventricular wall contact length (VVL) was defined as the percentage of the catheter’s 16 mm perforated tip that appeared to extend beyond the ventricular wall boundary in the 3D reconstruction based on CT data, with such protrusions interpreted as possible partial volume effects on imaging (Figure 4D).

For time assessment, intraoperative procedural time was defined separately for the two methods in both *ex vivo* and *in vivo* studies. For PS-3DGS, it was measured from initial guide placement to stylet removal, including burring and catheter insertion, but excluded the preoperative design and printing processes. For EM neuronavigation, it was measured from the start of navigation synchronization to stylet removal, encompassing surgical planning, burring, and catheter insertion, but excluding preoperative fiducial placement and CT scanning. These times were recorded with a stopwatch for each trial to allow comparison of intraoperative procedural duration between methods.

Finally, to evaluate the influence of soft tissue on guide performance and to validate the transferability of the *ex vivo* model to clinical conditions, accuracy outcomes from the *in vivo* PS-3DGS group were statistically compared with those from the five *ex vivo* Beagle CVMs. Similarly, the accuracy of neuronavigation-guided placements was compared between *in vivo* and *ex vivo* settings to assess whether real surgical conditions affected the precision of the navigation system.

### 2.9 Statistical analysis

All data were processed using SPSS version 26 (IBM SPSS, Chicago, IL, USA). Continuous variables were tested for normality using the Shapiro–Wilk test. For normally distributed data, mean and standard deviation were reported and paired comparisons were conducted using a paired t-test. For non-normally distributed data, median and interquartile range (IQR) were reported and the Wilcoxon signed-rank test was used. In the *ex vivo* study, paired comparisons were performed between PS-3DGS and EM neuronavigation methods for all quantitative metrics. Pearson’s correlation analysis was conducted to evaluate relationships between anatomical variables and catheter placement accuracy. In the *in vivo* study, comparisons between the two methods were also conducted using the Wilcoxon signed-rank test due to small sample size and non-normal data distribution. Correlations between anatomical variables and placement accuracy were examined using Spearman’s rank correlation. To compare the results of *in vivo* and *ex vivo* placements within the same specimens, a Mann–Whitney U test was applied, treating the groups as independent. *A priori* power analysis for the *in vivo* study was conducted using G*Power (v3.1.9.7, Heinrich Heine University Düsseldorf, Germany) with parameters set at α = 0.05, power = 0.8, and an effect size derived from *ex vivo* results, which indicated a minimum of five dogs. A *post hoc* power analysis was also performed for the *ex vivo* study’s primary outcome (paired t-test for DFM) to confirm the adequacy of the sample size (n = 10 placements per method). A significance level of *p* < 0.05 was considered statistically significant for all analyses.

## 3 Results

### 3.1 *Ex vivo* study

All 20 ventricular catheter placements in the cranio-ventricular models were completed without procedural failures. Both the PS-3DGS and EM navigation systems successfully guided the catheter tip into the intended lateral ventricle in every trial. The dogs used for CVM generation exhibited a representative range of skull morphologies, with an overall SI of 66.1 ± 11.1 and CI of 71.5 ± 11.2. Based on the classification criteria described in a previous study (28), skulls with an SI < 51 were classified as dolichocephalic, those with 51 ≤ SI < 59 as mesocephalic, and those with SI ≥ 59 as brachycephalic. In this study, the Beagle dogs had SI values ranging from 53.97 to 59.75 (median 56.45), were predominantly classified as mesocephalic, although some values are close to the brachycephalic threshold. Pomeranians, with SI values between 69.52 and 83.10 (median 76.65), and Maltese, with an SI of 72.92, were classified as brachycephalic. The median VBR in the models used for PS-3DGS and neuronavigation placements were 23.51% (IQR 10.96–26.18%) and 27.29% (IQR 12.47–29.08%), respectively. The reproducibility of lateral ventricles was 93.05 ± 4.66%. The synchronize accuracy was 0.844 ± 0.25 mm. The mean navigational registration error was 0.84 ± 0.25 mm, reflecting accurate image-to-specimen alignment.

The planned optimal trajectory had a DFM of 5.32 ± 1.52 mm. The PS-3DGS achieved a DFM of 5.56 ± 1.45 mm, and the neuronavigation placements had DFM 5.54 ± 2.09 mm in the models. There was no statistically significant difference in between the PS-3DGS and the planned trajectory (*p* = 0.438), nor between PS-3DGS and neuronavigation (*p* = 0.981). The median differences in X, Y, and Z coordinates between the actual PS-3DGS tip and the planned tip were not significant (∆X: 0.92 mm, ∆Y: 0.02 mm, ∆Z: −0.6 mm; *p* = 0.333, 0.646, 0.646 respectively; Table 1). The tip placement error and entry point error for PS-3DGS were a 2.81 ± 1.06 mm and 5.41 ± 1.42 mm, respectively. IIL and VVL were evaluated as measures of how well each method positioned the catheter within the ventricle. The median IIL in the PS-3DGS group was 18.55 mm (IQR 16.75–19.18 mm) and in the neuronavigation group was 22.35 mm (IQR 19.63–25.53 mm). By design, the perforated section of the catheter is 16 mm; thus, these values indicate that in many cases the catheter went slightly beyond the ventricle. The neuronavigation group had a significantly greater IIL than the PS-3DGS group (*p* = 0.028), meaning the navigated catheters tended to penetrate deeper into the ventricle (or even through to the far side) compared to the guide placements. However, when expressed as a percentage of the 16 mm perforated segment, both groups had median IIL ratios at or above 100%. In all cases, at least some portion of the perforated catheter was intra-ventricular. With respect to ventricular wall contact, the PS-3DGS showed a clear advantage. In the PS-3DGS group, the median VVL was 0% (IQR 0–35.2%), meaning in over half of the guided placements, the perforated VC part was entirely free-floating in the ventricle with no wall contact. In contrast, the neuronavigation group had a median VVL of 43% (IQR 23–48.31%), indicating that typically nearly half of the perforated section was pressed against the ventricle lining. This difference was statistically significant (*p* = 0.011) (Figure 4).

**Table 1:** Comparison of the tip coordinates between the plan and PS-3DGS groups (*ex vivo*).

Coordinates		X	Y	Z
Plan	Median	−0.561	0.168	2.256
	IQR	−1.338-0.36	−3.956-4.284	1.759–3.051
PS-3DGS	Median	−1.483	0.337	2.917
	IQR	2.286–1.188	−2.619-3.858	1.184–4.753
*p*-value		0.333	0.646	0.646

The mean intraoperative procedural time for PS-3DGS was 244.8 ± 76.3 s (approximately 4 min), compared to 520.4 ± 159.3 s (approximately 8.7 min) for the neuronavigation method. This nearly two-fold decrease in intraoperative procedural time with PS-3DGS was statistically significant (*p* = 0.039). Correlation with Anatomical Variables: Pearson correlation analysis in the PS-3DGS group revealed a notable relationship between skull shape and entry accuracy. There was a significant negative correlation between the entry point error (for PS-3DGS placements) and the SI of the model (r = −0.637, *p* = 0.048). This indicates that models with higher SI (i.e., more brachycephalic skull type) tended to have smaller entry point deviations. No significant correlation was found between ventricle size (VBR) and any accuracy metric, nor between CI and accuracy. A *post hoc* power analysis for the *ex vivo* DFM comparison (paired t-test, effect size = 1.807, n = 10, α = 0.05, two-tailed) yielded a power of 0.999, confirming sufficient sample size.

### 3.2 *In vivo* study

All five Beagle dogs tolerated the surgical procedures without any major intraoperative or postoperative complications. The median CI and SI values measured from the CT scans were 60.54 (IQR 58.398–64.904) and 56.45 (IQR 55.283–56.51), respectively, which fall within the range consistent with a mesocephalic skull type. The median VBRs for the PS-3DGS and neuronavigation groups were 10.84% (IQR 10.5–11.31%) and 12.46% (IQR 9.99–12.51%), respectively. The median synchronization accuracy was 1.3 mm (IQR 1.2–1.6 mm). Both the neuronavigation and PS-3DGS methods were successfully employed in each dog, with a total of 10 catheters placed (5 with each method).

The median DFM was 5.86 mm (IQR 5.10–6.42 mm) for the planned trajectories, 6.11 mm (IQR 5.96–7.19) for PS-3DGS, and 5.92 mm (IQR 3.13–6.59) for neuronavigation. There were no significant differences in DFM between the PS-3DGS and the plan (*p* = 0.686), nor between PS-3DGS and navigation (*p* = 0.345). In terms of 3D coordinates, the guide vs. plan comparison showed no significant offsets (median ∆X = 0.87 mm, ∆Y = 1.68 mm, ∆Z = 1.14 mm for PS-3DGS relative to plan; *p* = 0.225, 0.893, 0.345, respectively; Table 2). The tip placement error and entry point error for PS-3DGS were a 6.16 mm (IQR 3.04–6.63 mm) and 4.85 mm (IQR 4.57–6.81 mm), respectively. When we compared the *in vivo* guide performance to the *ex vivo* guide performance, most accuracy measures were statistically indistinguishable. In particular, the DFM, tip placement error, entry point error, and tip coordinates for PS-3DGS showed no significant differences between the beagle dogs and their corresponding models (all *p* > 0.05).

**Table 2:** Comparison of the tip coordinates between the plan and PS-3DGS groups (*in vivo*).

Coordinates		X	Y	Z
Plan	Median	0.497	4.009	2.563
	IQR	−0.053 ~ 0.928	−4.546 ~ 4.376	2.135 ~ 2.302
PS-3DGS	Median	−0.375	2.325	1.975
	IQR	−1.473 ~ 2.302	−1.375 ~ 3.953	0.995 ~ 2.013
*p*-value		0.225	0.893	0.345

Because the Beagle dogs had normal small ventricles, quantifying VVL was not feasible—often the ventricle collapsed around the catheter. Therefore, VVL results are not reported for *in vivo* placements. IIL was measurable in all cases, and each catheter successfully traversed the full extent of the ventricular lumen. The median IIL was 19.2 mm (IQR 19.0–23.1 mm) for PS-3DGS and 21.6 mm (IQR 18.6–27.7 mm) for neuronavigation, with no significant difference between methods (*p* = 0.50). All placements had IIL exceeding the 16 mm perforated tip length. In contrast to the *ex vivo* scenario, there is no significant difference in the intraoperative procedural time (p = 0.5). The median intraoperative procedural time with neuronavigation was 1,720 s (IQR 1,613–2,167 s), whereas for PS-3DGS it was 2,010 s (IQR 1,272–2,453 s).

Spearman correlation analysis in the experimental dog data revealed trends analogous to the model findings. There was a negative correlation between entry point error and the cranial index for the dogs (r = −0.900, *p* = 0.037). Additionally, the neuronavigation registration accuracy was found to correlate positively with DFM error (r = 0.900, p = 0.037).

#### 3.2.1 Postoperative imaging and complications

On the immediate postoperative CT scans, all guided and navigated catheter tips were confirmed to be within a ventricular space. Out of 10 placements, 8 catheters were located in the intended (ipsilateral) lateral ventricle on postoperative CT. In the remaining 2 cases, each method had one outlier: one PS-3DGS-guided catheter in one dog entered the third ventricle (instead of the lateral ventricle, presumably passing through the foramen of Monro), and one neuronavigated catheter in a different dog crossed into the contralateral lateral ventricle. Notably, neither of these misplacements caused neurological symptoms, and the catheters were still within CSF spaces.

**Figure 5:**
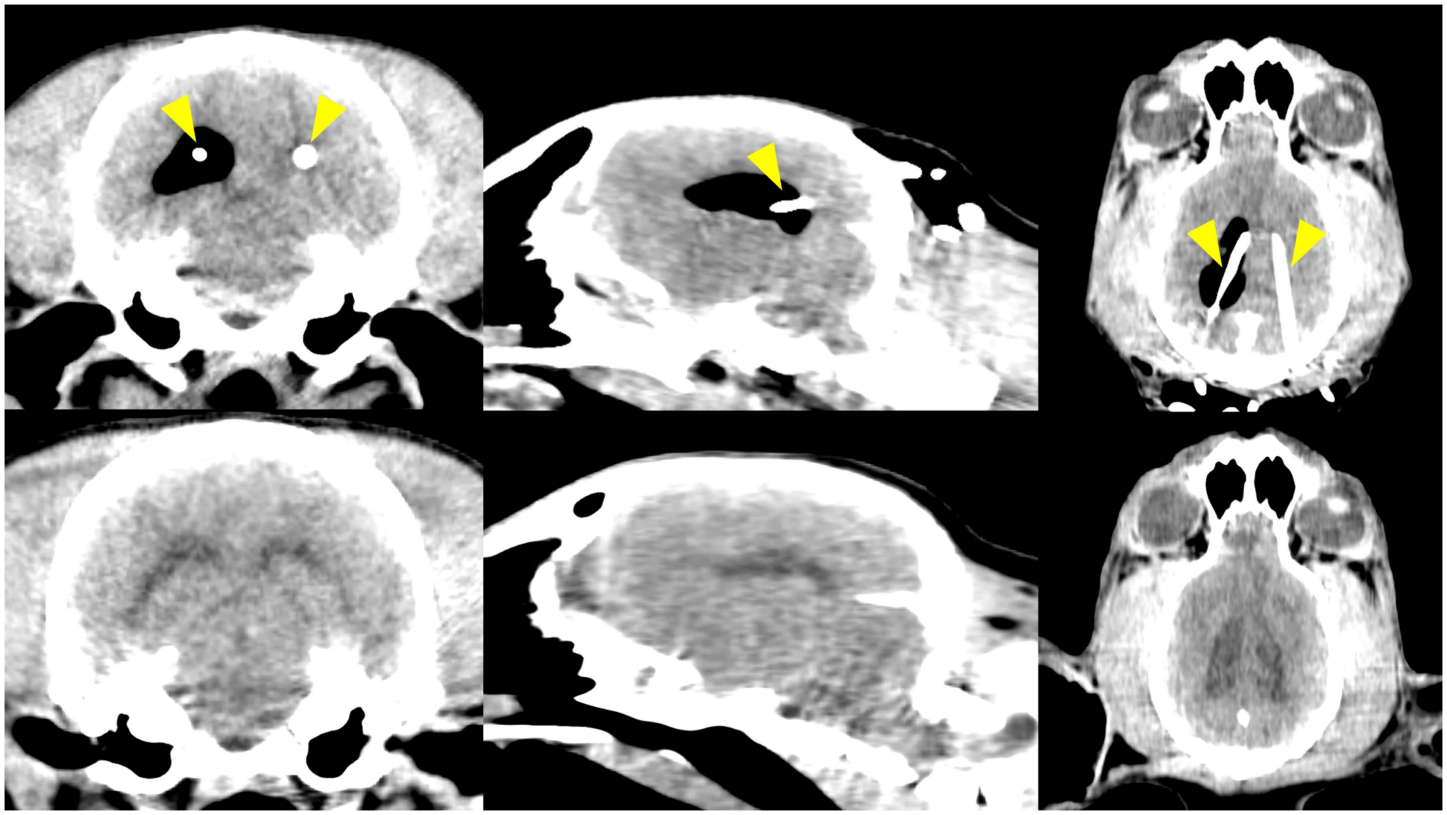
Pneumoventricle was observed as a postoperative complication in the first beagle. Top row: Immediate postoperative CT images (transverse, parasagittal, and dorsal views) show air in a mildly dilated right lateral ventricle, while the left ventricle is collapsed. The ventricular catheters are indicated by yellow arrowheads. Bottom row: CT images at 2 months post-surgery show resolution, with both lateral ventricles returned to normal size and no residual air.

A noteworthy finding was observed in one dog (Dog 1), which was the first subject to undergo the procedure. The right lateral ventricle appeared abnormally distended and filled with air (pneumoventricle), whereas the contralateral ventricle on the guide side was collapsed. Despite the pneumoventricle, the dog exhibited no clinical signs. A follow-up CT performed 2 months later confirmed complete resorption of the air and normalization of both ventricles, with no evidence of lasting damage (Figure 5). To prevent recurrence of this phenomenon in subsequent dogs, we adopted and modified a preventive maneuver from human neurosurgery (29). Immediately after placing the second catheter, a sterile 5 mL syringe half-filled with saline was attached to the catheter hub and held at the level of the tragus of the ipsilateral ear to approximate ventricular pressure. Gentle aspiration then allowed air to bubble into the syringe and saline to flow in gradually, with approximately 1 mL of saline replacing every 2 mL of air. This exchange was repeated until no further air emerged. Using this technique, no significant pneumoventricle occurred in the remaining dogs. A small pocket of air was noted in one case, but it was asymptomatic and resolved spontaneously. This straightforward air–saline exchange technique, adapted from human neurosurgical protocols, proved effective in our series.

Aside from this transient complication, there were no infections, hemorrhages, or neurologic deficits observed in any dog during 14 days of postoperative monitoring. All surgical incisions healed without complication.

## 4 Discussion

In this study, we evaluated a PS-3DGS for VC placement in dogs and found that its performance was comparable to that of EM neuronavigation in both laboratory and experimental surgical settings. The PS-3DGS achieved high placement accuracy, consistently targeting the ventricle as effectively as EM navigation. In the *ex vivo* model, the guided placements closely matched the planned optimal trajectory and showed no significant differences in tip placement or entry point errors relative to neuronavigation. Similarly, *in vivo*, the guided approach yielded catheter positions virtually indistinguishable from those achieved with navigation. These findings support our hypothesis that a well-designed 3D guide can replicate the precision of modern navigation systems. PS-3DGS achieved accurate targeting and successful ventricular entry in all cases, supporting its feasibility and reliability.

DFM was selected as the primary accuracy metric because it directly reflects how closely the catheter approaches the ideal target for CSF drainage. In human neurosurgery, the foramen of Monro is widely considered the optimal tip location for ventricular catheter placement, maximizing CSF outflow and reducing the risk of occlusion (13, 30, 31). Aligning the catheter tip with this landmark ensures the perforated segment is well positioned within the CSF-filled space. Prior studies have similarly used DFM as a benchmark for assessing catheter accuracy (32). In our study, the guide design explicitly aimed to minimize DFM, and the PS-3DGS consistently achieved DFM values equivalent to both the preoperative plan and neuronavigation, validating its targeting precision. Thus, DFM serves as a clinically meaningful and quantifiable indicator of placement accuracy, justifying its use as the primary outcome metric. The.

In addition to targeting accuracy, we assessed functional catheter positioning using two metrics: IIL and VVL. IIL quantifies how much of the catheter’s perforated segment resides within the ventricle. Insufficient IIL—where holes remain in parenchyma or contact the ventricular wall—is a known risk factor of shunt obstruction (18, 33). Therefore, IIL over 16 mm would be considered as optimal length of intraventricular placement. VVL measures how much of the perforated tip contacts the ventricular wall, which can obstruct apertures and compromise flow. High VVL may be associated with poor shunt function, which is due to the obstruction by the choroid plexus or ependymal tissue. In these cases, the need for revision surgeries in both human and veterinary studies has reported (12, 14, 15, 34). In our *ex vivo* results, PS-3DGS achieved significantly lower VVL than neuronavigation, suggesting a more favorable free-floating position of the catheter within the CSF. Although VVL could not be assessed *in vivo* due to collapsed ventricles, all placements ensured the perforated segment was entirely within the ventricle. Together, IIL and VVL complement DFM by evaluating not only whether the catheter reached its target, but also how optimally it was positioned to sustain long-term function. These functional parameters provide a deeper understanding of the clinical relevance of catheter placement beyond geometric accuracy. However, some human studies have reported that neither the length of catheter insertion nor its contact with the ventricular wall were significant risk factors for complications (35, 36). This indicates that while IIL and VVL provide valuable insights into functional catheter positioning, their direct impact on veterinary clinical outcomes has yet to be fully established.

In the electrode study, the overall mean placement error was 4.6 ± 1.5 mm when targeting the anterior thalamus and hippocampus using a frameless stereotactic system (37). In another study using a 3D guide for brain biopsy, stereotactic accuracy was higher, with a mean error of 1.68 mm (range 0.82–3.16 mm) (38). In our study, the tip placement error was 2.81 ± 1.06 mm in the *ex vivo* phase and 6.16 mm (IQR 3.04–6.63 mm) in the *in vivo* phase, values somewhat larger than those reported in previous stereotactic studies. However, unlike those studies, the accuracy of ventricular catheter placement should not be judged solely by the stereotactic precision of the tip position. Importantly, we adopted DFM as the primary accuracy metric, which did not differ significantly between the planned and neuronavigation groups, and all perforated catheter segments were consistently located within the ventricles. Taken together, although the stereotactic accuracy of tip placement in our study appeared lower, the overall accuracy of ventricular catheter placement—when assessed using multiple functional parameters such as DFM, entry point error, IIL, and VVL—was comparable to that of the planned and neuronavigation groups. Moreover, considering that clinical hydrocephalic patients typically present with ventriculomegaly, the applicability of PS-3DGS in practice may be even more favorable.

Statistical correlation analysis revealed that anatomical characteristics influenced entry point accuracy. Initially, it was hypothesized that a lower SI representing a more dolichocephalic skull type, would provide a broader contact surface between the midline guide and the skull, thereby improving placement accuracy. However, Pearson correlation analysis during the CVM phase revealed a potential inverse relationship between SI and entry point error, i.e., less entry point error with higher SI. This finding may be explained by breed-specific skull morphology, as brachycephalic dogs tend to have shorter skulls (higher CI and SI) that result in a shorter midline guide, thereby reducing guide-related deformation—such as structural flexibility or minor distal distortion—and contributing to increased placement precision. Unlike the CVM, which included both brachycephalic and mesocephalic breeds, all subjects in the *in vivo* phase were Beagles. In this context, the *in vivo* analysis demonstrated a negative correlation between entry point error and the CI, rather than SI. This may be due to the relatively narrow SI range among Beagles, making individual differences in CI more influential on guide stability. Furthermore, the decreased placement accuracy may be attributed not only to the longer guide span but also to interference from overlying soft tissues. Notably, soft tissue over the cranial vault, such as the temporal muscles, is generally thicker than that over the nasal and frontal bones. This discrepancy in tissue thickness may affect the stability and positioning of the guide during surgery. These findings are consistent with previous studies suggesting that the inclusion of soft tissue can reduce guide accuracy (23). From a clinical perspective, PS-3DGS may be particularly well suited for brachycephalic breeds, which are not only predisposed to hydrocephalus but may also benefit from the increased mechanical stability afforded by their skull morphology and less interference from soft tissue around the calvarium.

While the PS-3DGS performed well, our experience identified a few areas for potential refinement in the guide’s design and usage. In the *ex vivo* phase, the PS-3DGS group demonstrated a significantly shorter procedural time compared to EM neuronavigation (*p* = 0.039). However, this advantage was not observed in the *in vivo* phase, where no significant difference between the two methods was found (*p* = 0.5). This discrepancy was likely attributable to the additional time required in actual surgery to maintain sterility at the surgical site while attaching the anchor guide, as well as the considerable time needed to maneuver the skin for accurate placement of the gliding section. One key improvement is to increase the maneuverability of the guide system to reduce intraoperative procedural time and enhance ease of use. Specifically, we propose modifying the guide into a modular configuration: the current single-piece midline guide could be divided into two components—a fixed main guide and a detachable gliding sleeve. In practice, the main midline guide would be secured in position, and the secondary gliding guide could then be attached or detached from the caudal end of the main guide as needed during burring. This would eliminate the cumbersome need to repeatedly remove and reposition the entire guide when verifying the entry point or burring depth. A second related refinement is the creation of interchangeable gliding guide inserts tailored to different tissue layers. We envision having one insert contoured to slide smoothly over the skin surface and another insert shaped to interface directly with the skull once a small skin incision is made. The skin-surface insert could be used for initial positioning and marking of the entry on an intact scalp, after which it could be swapped for the bone-contact insert to guide the drill bit precisely into the cranium. By having these two options, the surgeon can maintain continuous guided alignment from skin marking through craniotomy without repeatedly lifting the entire guide. These modifications, including a two-part guide and dual-layer inserts, are intended to streamline the workflow and reduce PS-3DGS setup and intraoperative procedural time, thereby making the system more user-friendly and clinically practical through targeted design refinements.

One notable perioperative complication encountered in our *in vivo* study was a pneumoventricle. This occurred in the first dog when catheters were placed sequentially in both lateral ventricles. Although this phenomenon is rare in veterinary medicine and scarcely reported in the literature (39), it is a recognized complication in CSF diversion procedures (29, 40). In our case, the left lateral ventricle—cannulated first—appeared collapsed on post-placement imaging, while the subsequently placed catheter in the right ventricle revealed that it was dilated and filled with air. The exact mechanism remains unclear, but we suspect that CSF drainage from the first ventricle reduced intraventricular pressure and volume, causing the ventricle to shrink. When the second shunt was inserted contralaterally, the resulting pressure gradient may have drawn air into the ventricular system through the shunt tubing, leading to an air-filled ventricle on one side. Fortunately, the pneumoventricle was promptly recognized on immediate postoperative CT. To prevent recurrence, we adapted a technique described in human neurosurgery, originally developed to relieve tension pneumoventricle (29). This adjustment effectively prevented recurrence in subsequent cases. The dog recovered without any neurological deficits, and follow-up imaging at 2 months confirmed normalization of both ventricles with complete resorption of intracranial air. While pneumoventricle is uncommon, this experience highlights the importance of post-placement verification imaging and the need to remain prepared for unexpected shunt-related complications, even in controlled experimental settings.

The trajectory chosen for ventricular catheter placement in this study was conceptually inspired by the Frazier’s point approach commonly used in human neurosurgery. In human patients, the Frazier’s point entry—located in the posterior parietal region—provides a straight path into the anterior horn of the lateral ventricle and is a well-established freehand technique for shunt insertion (41, 42). Our PS-3DGS planning similarly targeted an entry on the caudal dorsolateral cranium (parietal bone) with the catheter directed caudo-rostrally to lie along the ventricular body. This results in the catheter tip residing near the foramen of Monro and the catheter shaft coursing within the ventricle, analogous to the endpoint of the Frazier’s technique in humans. However, despite this resemblance, we discovered that a one-size-fits-all craniometric approach is not feasible in dogs. Canine ventricular anatomy is highly variable: even within the same dog, the left and right lateral ventricles can differ in size and shape, and across breeds there is an even greater diversity in cranial morphology. Our computer modeling and trajectory simulations revealed that the optimal entry and target points differed markedly between contralateral ventricles and between individuals. Unlike in humans, dogs do not have a single standardized entry that guarantees ventricular access due to the wider range of skull shapes and ventricle configurations. This finding supports the premise that a tailored, patient-specific approach is needed for accurate ventricular catheter placement in veterinary patients. PS-3DGS allows such customization by planning the trajectory on each dog’s CT and designing the guide to fit that dog’s anatomy, we effectively compensate for individual anatomical variations that would challenge any uniform technique. The success of our guided placements affirms that custom trajectory planning is a viable solution to the variability problem, providing each patient with a trajectory optimized for its unique anatomy.

There are several limitations to our study that must be acknowledged when interpreting the results. First, the experimental *in vivo* trial was conducted on healthy adult beagle dogs with normal ventricular anatomy. While this allowed us to establish baseline accuracy in a controlled scenario, it does not fully represent the scenario in clinical patients. Hydrocephalic dogs typically have greatly enlarged ventricles and potentially thinner cortical tissue, which could affect catheter placement dynamics and complication rates. Because our subjects had small, slit-like ventricles, certain functional assessments—such as measuring the exact VVL *in vivo*—were not feasible. Moreover, using normal dogs meant we could not evaluate how PS-3DGS might influence clinical outcomes like ventricle decompression, shunt patency over time, or improvement in neurological signs. Future studies in actual hydrocephalus patients are needed to determine whether the high placement accuracy of PS-3DGS translates into lower shunt failure rates or better patient prognoses. Second, the sample size was small. This sample was sufficient for detecting large differences in accuracy as confirmed by our power analysis, but a larger cohort would provide more robust statistical power and would allow assessment of variability across different sizes and breeds of dogs. All our subjects were of the same breed, which limits generalizability; canine skull shapes vary widely from brachycephalic to mesocephalic, and these extremes were not represented in our *in vivo* study. Third, there was a fixed order of method application in our design; neuronavigation was always performed before PS-3DGS in each animal. A counterbalanced or randomized order would have been ideal, though we adopted this sequence to avoid any loss of accuracy in the navigation system. However, this fixed order could introduce bias. It is possible that the act of placing the first shunt altered the intracranial environment slightly by draining some CSF or causing minor brain displacement in a way that influenced the subsequent guided placement on the opposite side. We tried to mitigate this by alternating which side was done with which method, but nonetheless the second procedure in each dog was not under identical conditions as the first. Fourth, the PS-3DGS technique requires preoperative planning and fabrication, which inherently demands additional time and resources before the surgery. In an acute emergency situation such as a rapidly deteriorating hydrocephalus patient, waiting to obtain a CT scan, design a guide, and 3D-print the device could delay treatment. In our study, all dogs had a pre-existing CT scan that was used for guide creation, and the printing was done in advance. This workflow may not be readily available in all clinics. However, as 3D printing technology advances, production times and costs are decreasing, and patient-specific guides could potentially be made on short notice. Still, the need for preoperative imaging and guide fabrication is a limitation to consider when comparing PS-3DGS to real-time systems like neuronavigation. Finally, our evaluation of outcomes focused on accuracy and technical feasibility rather than long-term functionality. Since the dogs were not followed long-term with functioning shunts. Therefore, we cannot directly comment on whether PS-3DGS placement leads to fewer obstructions or prolonged shunt survival compared to neuronavigation. The design features of PS-3DGS are intended to reduce such complications, but a clinical trial in hydrocephalic dogs with follow-up would be necessary to validate this potential benefit. Further studies that address these limitations will be important to fully establish the utility of PS-3DGS for VPS in veterinary neurosurgery.

In conclusion, our findings support the clinical potential of PS-3DGS as a low-cost, customizable alternative to neuronavigation for accurate VC placement in dogs. Its accuracy, particularly in patients with brachycephalic anatomy, and potential for reduced wall contact highlight its utility in improving long-term shunt outcomes. Further clinical studies in hydrocephalic dogs are warranted to confirm these benefits and refine guide design for broader application.

## Data Availability

The raw data supporting the conclusions of this article will be made available by the authors, without undue reservation.

## Supplementary material

SUPPLEMENTARY TABLE S1 Coordinates and calculated results based on the Euclidean method.
